# Respiratory explants as a model to investigate early events of contagious bovine pleuropneumonia infection

**DOI:** 10.1186/s13567-017-0500-z

**Published:** 2018-01-12

**Authors:** Giovanni Di Teodoro, Giuseppe Marruchella, Andrea Di Provvido, Gianluca Orsini, Gaetano Federico Ronchi, Anna Rita D’Angelo, Nicola D’Alterio, Flavio Sacchini, Massimo Scacchia

**Affiliations:** 1 OIE Reference Laboratory for Contagious Bovine Pleuropneumonia, Istituto Zooprofilattico Sperimentale dell’Abruzzo e Molise “G. Caporale”, Campo Boario, 64100 Teramo, Italy; 2 Faculty of Veterinary Medicine, University of Teramo, Loc. Piano d’Accio, 64100 Teramo, Italy

## Abstract

Contagious bovine pleuropneumonia (CBPP) is a severe disease caused by *Mycoplasma mycoides* subsp. *mycoides* (*Mmm*). Knowledge on CBPP pathogenesis is fragmented and hampered by the limited availability of laboratory animal and in vitro models of investigation. The purpose of the present study is to assess respiratory explants as useful tools to study the early stages of CBPP. Explants were obtained from trachea, bronchi and lungs of slaughtered cattle, tested negative for *Mycoplasma* spp. and for the major bacterial and viral respiratory pathogens. The interaction of *Mmm* with explant cells was studied by immunohistochemistry (IHC), double-labelling indirect immunofluorescence (DLIIF) and laser scanning confocal microscopy (LSCM). *Mmm* capability to survive and proliferate within the explants was evaluated by standard microbiological procedures. Finally, the putative cellular internalization of *Mmm* was further investigated by the gentamicin invasion assay. IHC and DLIIF indicated that *Mmm* can colonize explants, showing a marked tropism for lower airways. Specifically, *Mmm* was detected on/inside the bronchiolar and alveolar epithelial cells, the alveolar macrophages and the endothelial cells. The interaction between *Mmm* and explant cells was abolished by the pre-incubation of the pathogen with bovine anti-*Mmm* immune sera. *Mmm* was able to survive and proliferate in all tracheal, bronchial and lung explants, during the entire time course of the experiments. LSCM and gentamicin invasion assay both confirmed that *Mmm* can enter non-phagocytic host cells. Taken together, our data supports bovine respiratory explants as a promising tool to investigate CBPP, alternative to cattle experimental infection.

**Electronic supplementary material:**

The online version of this article (10.1186/s13567-017-0500-z) contains supplementary material, which is available to authorized users.

## Introduction

Contagious bovine pleuropneumonia (CBPP) is a severe disease caused by *Mycoplasma mycoides* subsp. *mycoides* (*Mmm*) and represents a serious transboundary threat for livestock [1, 2]. CBPP is currently widespread in sub-Saharan Africa, where it is considered of major economic relevance through decreased animal productivity and the high cost of the control measures [3, 4].

Although *Mmm* can be detected in different body fluids and tissue types, the infection spreads through inhalation of droplets from diseased coughing animals [5]. CBPP clinical signs and pathological features are deeply influenced by several factors (e.g. host breed and age, strain virulence, stage of the disease) and mainly consist of respiratory distress and fibrinous pleuropneumonia, respectively [5, 6].

The pathogenesis of CBPP is considered multifactorial but remains largely unknown [7–9]. The capsular polysaccharide seems to promote *Mmm* adhesion to the host cells, enhancing the resistance to phagocytosis and playing a cytopathic effect on blood vessels [6, 10, 11]. The production of reactive oxygen species (ROS), the respiratory burst in phagocytic cells and immune-mediated mechanisms are supposed to further contribute to *Mmm* pathogenicity [12–15].

In the absence of suitable laboratory animal models, the study of CBPP mainly relies on the experimental infection of cattle. This approach is very expensive, ethically debatable, and of little value for investigating the early stages of the disease [16, 17]. Cell cultures have been used to study the host–pathogen interaction [9, 13, 18–20]; however, they do not accurately mimic the microarchitecture of the bovine airways and still foster the development of alternative, eligible in vitro models of investigation.

Taking this into consideration, the present study aims to evaluate whether bovine respiratory explants (BREs) are able to support the colonization, survival and replication of *Mmm*, thus acting as a suitable model to understand the early stages of *Mmm* infection and CBPP pathogenesis.

## Materials and methods

### Selection of animals

Samples were collected from 18 to 24 months old, Marchigiana breed cattle, belonging to herds located in the Abruzzi region (Central Italy) and officially free from CBPP, tuberculosis, brucellosis and bovine enzootic leukosis. Cattle were clinically healthy and regularly slaughtered, with no relevant lung lesions detected at post-mortem inspection.

Samples of trachea, bronchi, lung and tracheobronchial lymph nodes were collected from each animal and tested for *Mycoplasma* spp. [21], bovine viral diarrhea virus (BVDV) [22], bovine herpesvirus type-1 (BHV-1; VetMAX IBR gB, LSI), bovine respiratory syncytial virus (BRSV) and parainfluenza-3 virus (PI-3V; VetMAX Triplex BRSV & PI3, LSI) by polymerase chain reaction (PCR).

### Culture of bovine respiratory explants

At the slaughterhouse, the cranial portion of trachea, the accessory bronchus and the cranial lobe of the right lung were aseptically collected within 20 min from the stunning and bleeding of cattle. Samples were promptly immersed in transport medium (TM, Additional file 1), stored at 4 °C and delivered to the laboratory. BREs were obtained within 2 h from tissue collection, following a previously published protocol [23] with minor modifications.

#### Explants of the tracheal mucosa

The tracheal mucosa was stripped from the cartilage rings, cut in squares of about 25 mm^2^ and washed with fresh TM. Tissue samples were then transferred into 6-well plates, their bottom being covered with 2 mL of 1% agar gel (Biolife, Italy). The tracheal explants were cultured in an air–liquid interface system, slightly immersed in tissue culture medium (TCM, Additional file 1), with their epithelium facing up. The TCM was changed on a daily basis and the explants were incubated at 37 °C with 5% CO_2_ for up to 120 h.

#### Bronchial explants

The accessory bronchus was dissected from the lung parenchyma by means of a sterile scalpel and cut into pieces of about 25 mm^2^, without removing the cartilage. The bronchial samples were washed with fresh TM and cultured as reported above, using a specific TCM (Additional file 1).

#### Explants of lung parenchyma

The accessory bronchus was catheterized and the lung parenchyma embedded with 1% low gelling temperature agarose (type VII-A agarose, Sigma-Aldrich) at 37 °C. The lung lobe was then cooled to 4 °C for 20 min and cut into 1 mm thick slices. Tissue samples of about 25 mm^2^ were transferred into 6-well plates and maintained as for the other BREs, using a specific TCM (Additional file 1).

All BREs were tested daily for the presence of contaminant bacteria and fungi. To this aim, 100 µL of TCM per well were plated on 2% sheep blood agar and *Sabouraud* agar; culture plates were incubated at 37 °C and evaluated for growth after 72 and 96 h.

### Morphologic assessment of bovine respiratory explants

To assess their microscopic features, BREs were fixed in 10% neutral buffered formalin (NBF) at 0, 24, 48, 72, 96 and 120 h post-culture (hpc), embedded in paraffin and routinely processed for histology (hematoxylin and eosin stain, H&E). The distribution pattern of selected cellular markers was evaluated by immunohistochemistry (IHC), as detailed in Additional file 2. The microscopic morphology of BREs, as well as the IHC distribution pattern of the cell markers, were qualitatively evaluated by two independent investigators, blind to the experimental conditions. Control tissues (i.e. samples taken from the same animals at slaughtering and immediately fixed in 10% NBF) were used for comparison.

In addition, morphometric parameters (thickness of the tracheal and bronchial epithelium, extent of the alveolar walls) were measured in 10 high-power fields (final magnification = 400×) per time point per explant. Image analysis was carried out using *ImageJ* software (National Institutes of Health, Bethesda, USA) and data submitted for statistical analysis.

### *Mycoplasma mycoides* subsp. *mycoides*: strain, growth condition and titration

In the present study, we used a field strain of *Mmm*, which was isolated from a CBPP-affected cow in the Namibian region of Caprivi [24] and cultured in modified PPLO broth [25] at 37 °C with 5% CO_2_.

*Mmm* was titrated by serial tenfold dilutions, such assays being performed in duplicate. In detail, 100 µL of each dilution were plated on modified PPLO agar [25] and incubated at 37 °C with 5% CO_2_ for 72 h. The *Mmm* titre was expressed as colony-forming units (CFU)/mL.

### Challenge of bovine respiratory explants with *Mycoplasma mycoides* subsp. *mycoides*

BREs were challenged by immersion in 2 mL of TCM, which contained 2–6 × 10^8^ CFU/mL of *Mmm*. After 1 h of incubation, BREs were washed with PBS to remove non-attached *Mmm*, and cultivated in 6-wells plates up to 120 h post-inoculation (hpi). The TCM was changed on a daily basis. All infection assays were carried out in triplicate in three independent experiments and BREs were fixed in 10% NBF at 1, 24, 48, 72, 96 and 120 hpi. As above detailed, morphometric parameters (thickness of the tracheal and bronchial epithelium, extent of the alveolar walls) were measured in challenged BREs, using negative controls (i.e. non-infected BREs) for comparison.

An additional set of experiments were carried out to evaluate whether the interaction between *Mmm* and BREs might be inhibited by specific antibodies. To this aim, *Mmm* (2 mL of TCM containing 2–6 × 10^8^ CFU/mL) was preliminary incubated with bovine CBPP negative or positive serum (1 mL of OIE International Standard Serum for CBPP, complement fixation titer = 1/320) and then inoculated on lung explants, which were finally fixed in 10% NBF 1 hpi.

All 10% NBF-fixed samples were embedded in paraffin and routinely processed for H&E stain, IHC, double-labelling indirect immunofluorescence (DLIIF) and laser scanning confocal microscopy (LSCM) investigations.

### Immune detection of *Mycoplasma mycoides* subsp. *mycoides* in bovine respiratory explants

IHC was carried out following a recently published protocol [26]. Four µm-thick sections were mounted on positive charged glass slides, dried overnight at 37 °C, dewaxed and rehydrated using standard procedures. Antigen retrieval was performed by incubation with trypsin (Sigma-Aldrich; working solution = 0.01% in 0.15 M Tris–HCl buffer, pH 7.8) at 37 °C for 10 min. A murine monoclonal antibody anti-*Mmm* was used as primary antibody. Immune reactions were detected by means of a biotin-streptavidin amplification method and visualized using 3-3′-diaminobenzidine as chromogen (Dako REAL™ detection system). Lung samples from CBPP-naturally affected cattle which showed lesions at different stages of evolution (red-to-grey hepatization, necrosis, sequestra) acted as positive control. Negative controls consisted of CBPP negative lung samples and were also included in each IHC run.

DLIIF and LSCM investigations were carried out to better detail the cellular tropism of *Mmm*, both in challenged BREs and in samples collected from CBPP-affected cattle. To this purpose, tissue sections were incubated with primary antibodies anti-*Mmm*, lysozyme, von Willebrand factor (vWF) and cytokeratins (see Additional file 3 for details). Sections were mounted using a antifade medium with DAPI (Vector Laboratories, Inc.), stored at 4 °C in the dark until imaged using a Leica TCS SP5 II confocal microscope.

### Survival and growth of *Mycoplasma mycoides* subsp. *mycoides* in bovine respiratory explants

The *Mmm* ability to survive and grow within BREs was investigated in triplicate in 3 independent experiments. At different time-points (1, 24, 48, 72, 96 and 120 hpi), BREs were thoroughly rinsed in PBS and disrupted through high-speed shaking (30 Hz for 7 min) with stainless steel beads (Tissue Lyser II, Qiagen). The tissue homogenate (100 µL) was seeded in modified PPLO culture media and *Mmm* was titrated as above detailed. Culture media were considered negative in the absence of *Mmm* growth after 96 h of incubation.

The *Mmm* ability to survive and grow in TCM was also assessed, both in presence or in absence of BRE. To this aim, TCM (100 µL) was collected from each explant-containing well, plated on modified PPLO agar and evaluated for growth up to 96 h. In addition, *Mmm* (100 µL of PBS with 2–6 × 10^8^ CFU/mL) was seeded in 5 mL of each TCM (without explant), incubated up to 120 h, evaluated for growth and titrated as above described.

### Gentamicin invasion assay on lung parenchyma explants

The putative cellular internalization of *Mmm* was investigated in lung explants following gentamicin invasion assay protocols [27, 28], modified to fit our experimental conditions. Assays were performed in triplicate in two independent experiments.

Lung explants were inoculated with *Mmm* as above reported. Then, 24 hpi, explants were added with gentamicin (Sigma-Aldrich, 400 µg/mL of TCM) for 3 h, aiming to kill the extracellular *Mmm*. Thereafter, lung explants were washed with PBS, transferred in other 6-well plates to avoid the persistence of gentamicin in the agar gel, and cultured up to 120 hpi. The presence and the titer of *Mmm* were evaluated as above detailed, both in lung and TCM at 1, 24 (immediately before the gentamicin treatment), 27 (immediately after the gentamicin treatment), 48, 72, 96 and 120 hpi.

### Statistical analysis

Data were analyzed using a multivariate General Linear Model for repeated measures and are presented as mean ± standard deviations (SD). The differences were considered to be significant with *p* < 0.05. Statistical analysis was performed using the SPSS 15.0 software package (SPSS Inc. Chicago, IL, USA).

## Results

### Microbiological, histological and morphometric assessment of bovine respiratory explants

All cattle included in the present study tested negative for *Mycoplasma* spp., BVDV, BHV-1, PI-3 and BRSV.

The microscopical morphology of unchallenged BREs was considered satisfactory by both investigators (Additional file 4); the only relevant changes affected the mucosa-associated lymphoid tissue (MALT), which already at T24 showed massive apoptosis of lymphoid cells (Additional file 5).

**Figure 1:**
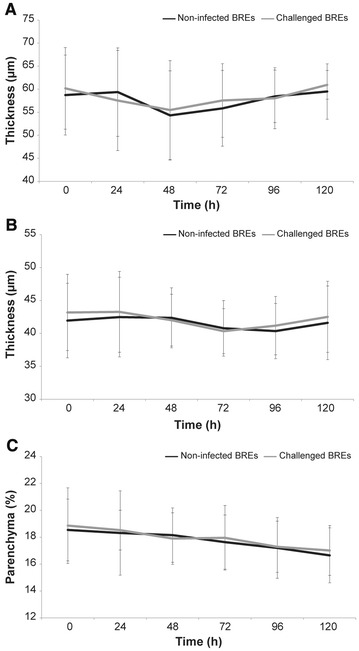
**Morphometric analysis of BREs.** The thickness of the unchallenged tracheal epithelium (**A**) was significantly reduced at 48 (*p ≤ 0.001) and 72 (**p = 0.01) hpc when compared with T0. However, in absolute terms, such differences were extremely small and always less than 5 µm. No significant difference was observed between non-infected and challenged tracheal explants (*p* ≥ 0.05). The thickness of the unchallenged bronchial epithelium (**B**) was not significantly modified along the entire time course of the experiment. No significant difference was observed between non-infected and challenged bronchial explants (*p* ≥ 0.05). The surface of the unchallenged alveolar walls—expressed as percentage of the entire field of observation—was not significantly modified along the entire the time course of the experiment (**C**). No significant difference was observed between non-infected and challenged lung explants (*p* ≥ 0.05). Data were represented as the mean ± SD.

The distribution pattern, intensity and specificity of cellular markers immunoreactivity (IR) appeared always well-preserved and resembled those observed in control tissues (Additional file 6). Morphometric parameters were overall stable during the entire time course of the experiments; the thickness of tracheal epithelium underwent very small reduction, while no significant change affected the thickness of bronchial epithelium and the extent of the lung parenchyma. The challenge with *Mmm* neither caused obvious pathological changes of BREs nor significantly modified any morphometric parameters (Figure 1).

### Immune detection of *Mycoplasma mycoides* subsp. *mycoides* in challenged bovine respiratory explants

**Figure 2:**
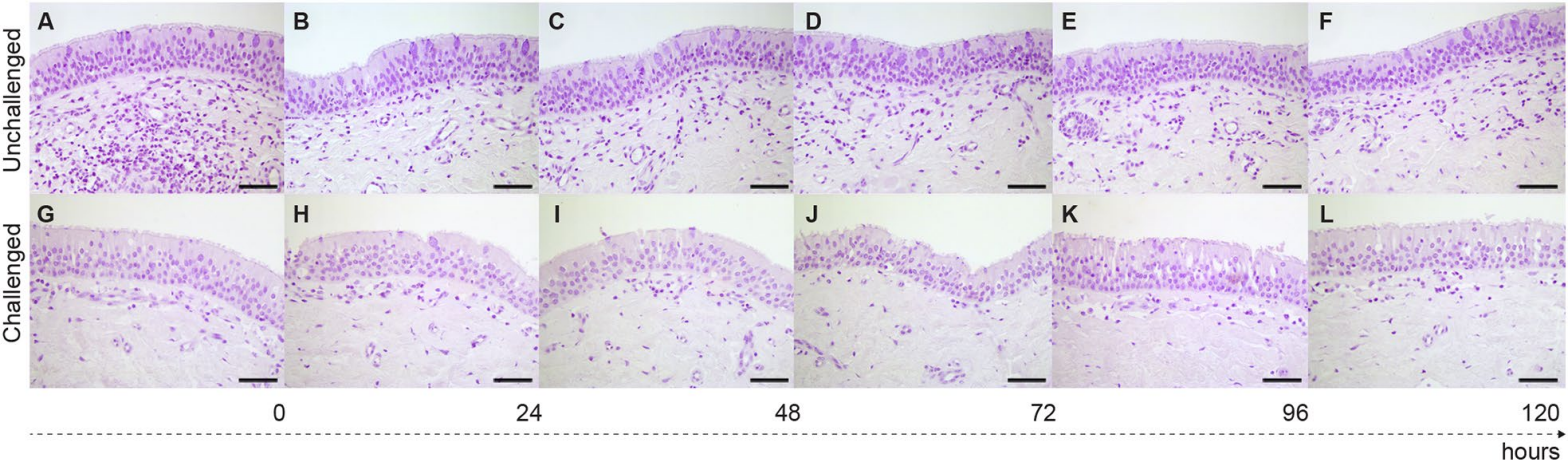
**Immunohistochemistry for** ***Mmm*** **on tracheal BREs.** No *Mmm*-IR was detected at the level of the tracheal epithelium and of the upper layer of the *lamina propria*, both in non-infected (**A**–**F**) and challenged (**G**–**L**) BREs. Mayer’s hematoxylin counterstain. Scale bar: 60 µm.

**Figure 3:**
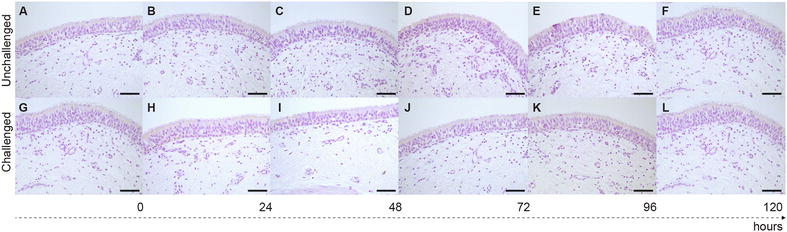
**Immunohistochemistry for** ***Mmm*** **on bronchial BREs.** No *Mmm*-IR was detected at the level of the bronchial epithelium and of the upper layer of the *lamina propria*, both in non-infected (**A**–**F**) and challenged (**G**–**L**) BREs. Mayer’s hematoxylin counterstain. Scale bar: 60 µm.

**Figure 4:**
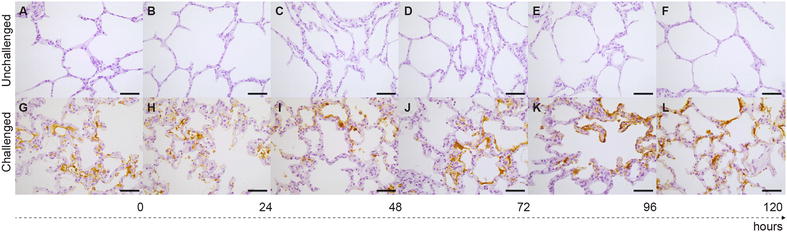
**Immunohistochemistry for** ***Mmm*** **on lung BREs.** No *Mmm*-IR was detected within the lung parenchyma of unchallenged BREs (**A**–**F**), while a specific *Mmm*-IR was clearly seen lining the alveolar walls of challenged BREs, during the entire time course of the experiment (**G**–**L**). Mayer’s hematoxylin counterstain. Scale bar: 60 µm.

**Figure 5:**
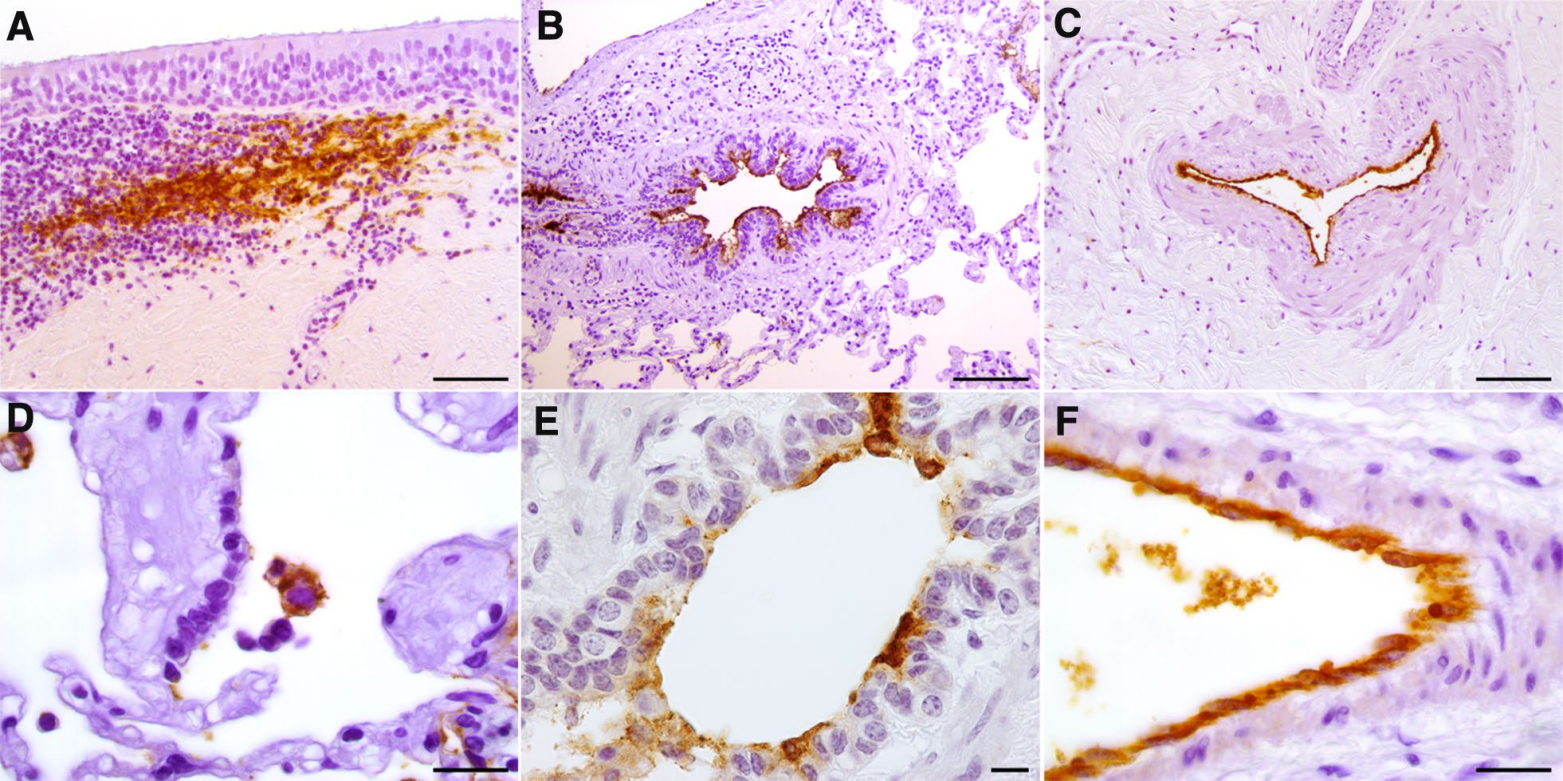
**Immunohistochemistry for** ***Mmm*** **on challenged BREs.** *Mmm*-IR was detectable within the MALT, the IHC pattern resembling that of the dendritic-cells (**A**; tracheal explant, T24). The presence of *Mmm* was observed upon the bronchiolar epithelium (**B**; lung explant, T1), along the endothelial surface of large blood vessel (**C**; tracheal explant, T24) and inside the cytoplasm of alveolar macrophages (**D**; lung explant, T1). At higher magnification, the *Mmm*-IR was apparently seen inside the cytoplasm of bronchiolar epithelial cells (**E**; lung explant, T1) and of endothelial cells (**F**; lung explant, T1). Mayer’s hematoxylin counterstain. Scale bar: 10 µm (**E**), 20 µm (**D**, **F**), 50 µm (**A**), 100 µm (**B**, **C**).

**Figure 6:**
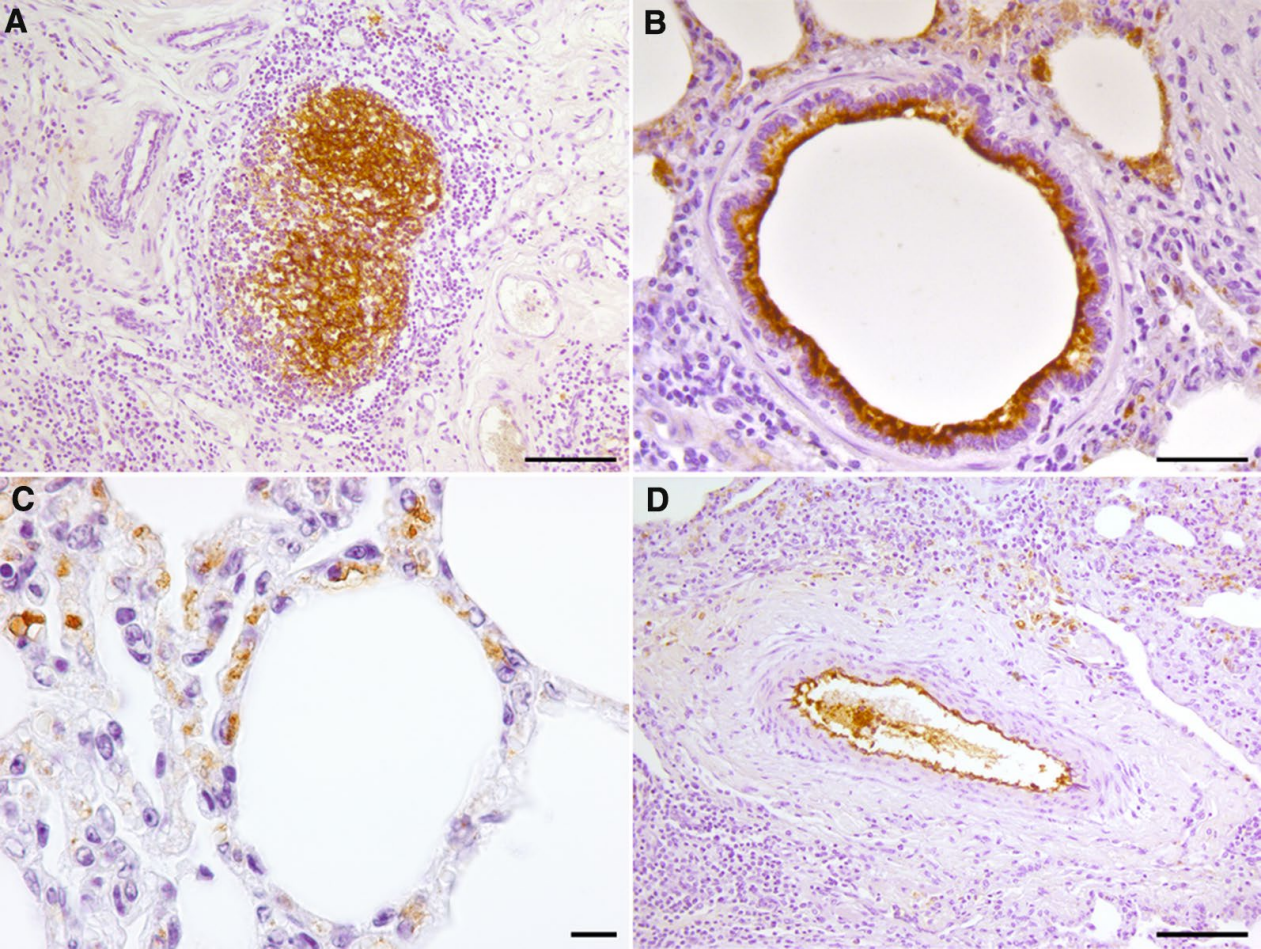
**Immunohistochemistry for** ***Mmm*** **in CBPP naturally affected cattle.** A specific, dendritic-like IR was observed in newly-formed lymphoid follicles, located within the wall of a sequestrum (**A**). The *Mmm*-IR was also detected along/inside the bronchiolar (**B**) and alveolar (**C**) epithelial cells, as well as along the endothelial surface of a blood vessel (**D**). Mayer’s hematoxylin counterstain. Scale bar: 10 µm (**C**), 20 µm (**B**), 50 µm (**A**, **D**).

**Figure 7:**
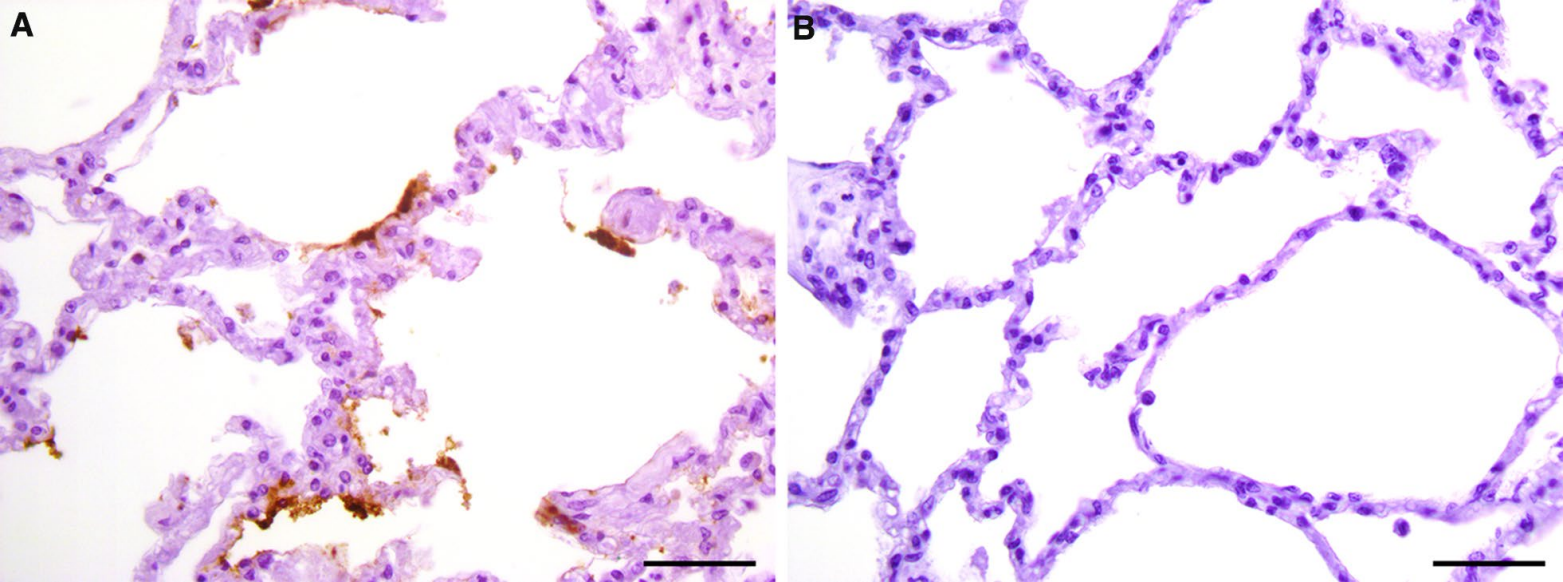
**Immunohistochemistry for** ***Mmm*** **after pre-incubation with bovine sera.** The pre-incubation of the pathogen with CBPP-negative serum did not modify the IHC pattern of *Mmm*, whose antigens were easily detected upon the alveolar surface (**A**). In contrast, *Mmm* antigens were undetectable in lung explant after the pre-incubation with a CBPP-positive serum (**B**). Mayer’s hematoxylin counterstain. Scale bar: 50 µm.

*Mmm*-IR largely varied among the different tracts of bovine airways. No specific IR was observed at the level of the tracheal and bronchial epithelium (Figures 2 and 3), except for small foci of disepithelialization, most likely due to tissue handling. In contrast, *Mmm* was consistently detected along and/or inside the bronchiolar epithelium and the alveolar cells, remaining apparently unchanged up to 120 hpi (Figures 4 and 5). Furthermore, *Mmm* was seen within the MALT showing a dendritic-like pattern, within the cytoplasm of the alveolar macrophages, as well as upon/within the endothelial cells from all BREs (Figure 5). In general, the *Mmm*-IR in lung explants closely resembled that observed in CBPP-affected cattle, which were used as positive controls (Figure 6). The interaction between *Mmm* and lung explants was dramatically reduced, if not completely abolished, by pre-incubation of the pathogen with anti-*Mmm* bovine immune sera (Figure 7).

**Figure 8:**
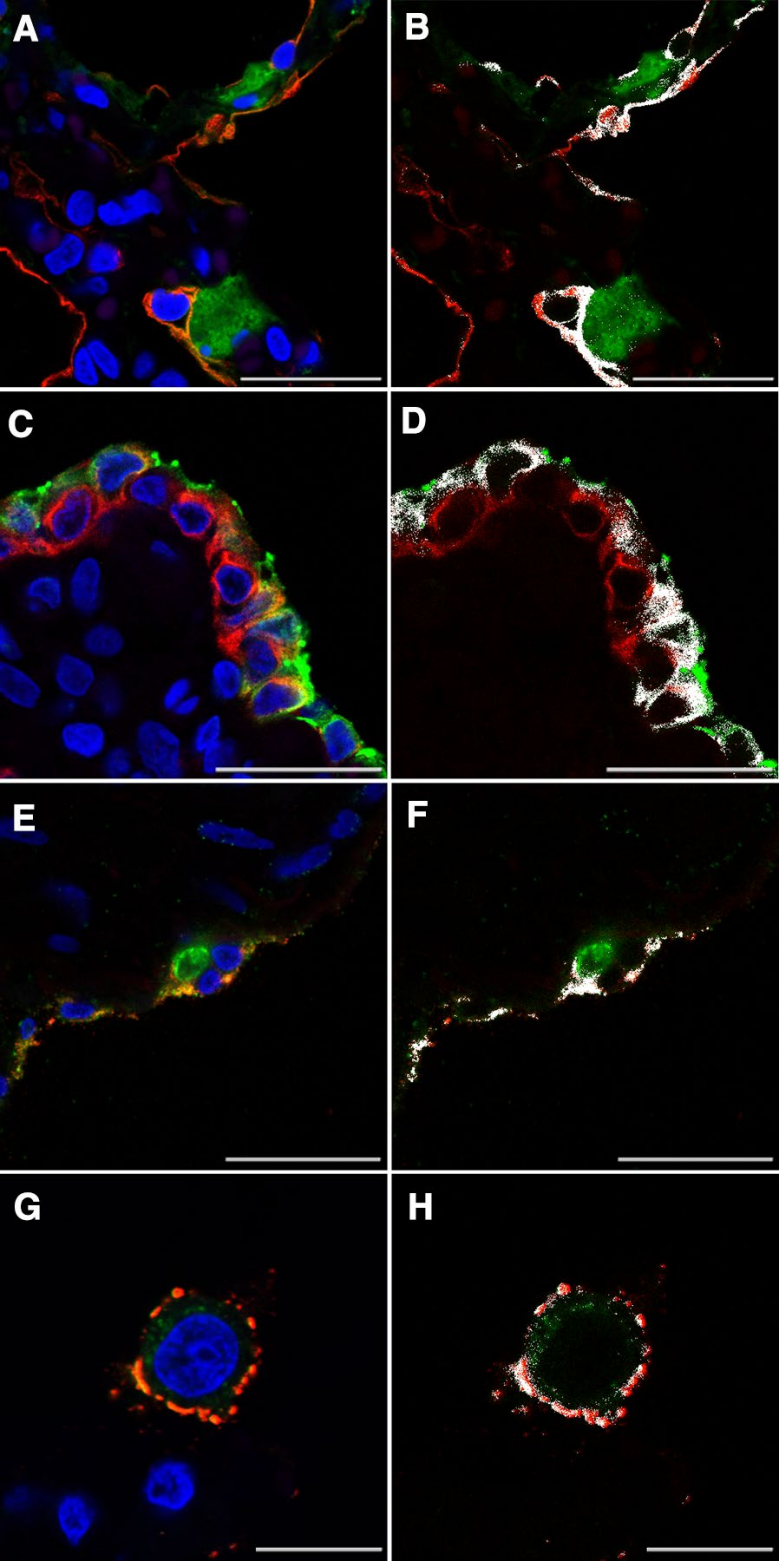
**Lung explants challenged with** ***Mmm*—laser scanning confocal microscopy. A** The merging of fluorochromes highlighted the close interaction between *Mmm* (green color) and pneumocytes (cytokeratin, red color). Colocalization of red and green signals was shown in white (**B**) and indicated the *Mmm* localization inside the cytoplasm of alveolar cells. **C** The merging of fluorochromes highlighted the close interaction between *Mmm* (green color) and the bronchiolar epithelium (cytokeratin, red color). Colocalization of red and green signals was shown in white (**D**) and indicated the *Mmm* localization inside the cytoplasm of bronchiolar cells. **E** The merging of fluorochromes highlighted the close interaction between *Mmm* (red color) and the endothelium (vWF, green color). Colocalization of red and green signals was shown in white (**F**) and indicated the *Mmm* localization inside the cytoplasm of endothelial cells. **G** The merging of fluorochromes highlighted the close interaction between *Mmm* (red color) and the alveolar macrophage (lysozyme, green color). Colocalization of red and green signals was shown in white (**H**) and indicated the localization of *Mmm* within cytoplasm of the alveolar macrophage. Nuclei were always stained in blue with DAPI. All pictures were kept at T24. Scale bar: 10 µm (**G**, **H**), 22.5 µm (**A**–**F**).

**Figure 9:**
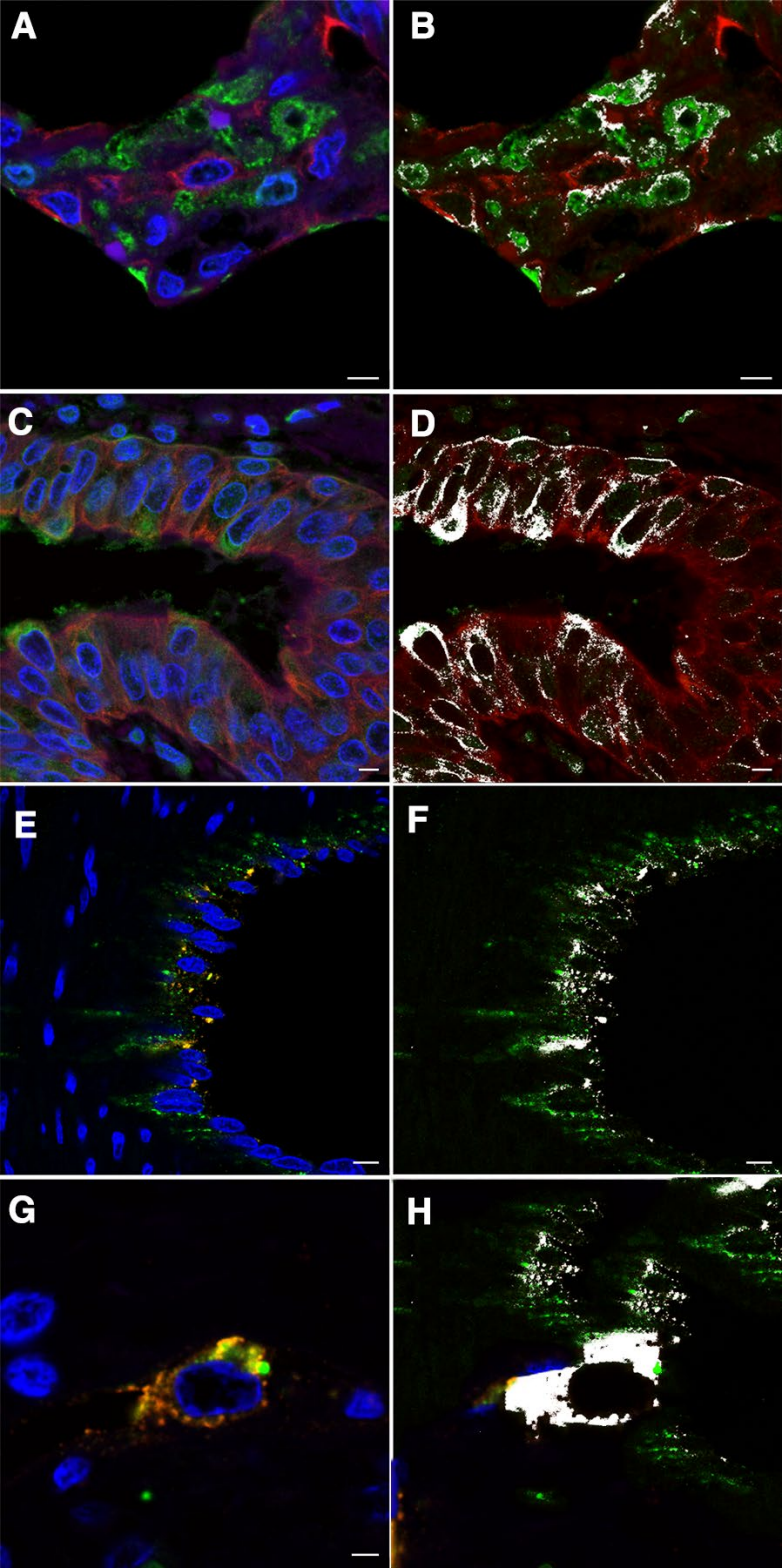
**Lung samples from CBPP naturally affected cattle—laser scanning confocal microscopy. A** The merging of fluorochromes highlighted the close interaction between *Mmm* (green color) and pneumocytes (cytokeratin, red color). Colocalization of red and green signals was shown in white (**B**) and indicated the *Mmm* localization inside the cytoplasm of alveolar cells. **C** The merging of fluorochromes highlighted the close interaction between *Mmm* (green color) and the bronchiolar epithelium (cytokeratin, red color). Colocalization of red and green signals was shown in white (**D**) and indicated the *Mmm* localization inside the cytoplasm of bronchiolar cells. **E** The merging of fluorochromes highlighted the close interaction between *Mmm* (red color) and the endothelium (vWF, green color). Colocalization of red and green signals was shown in white (**F**) and indicated the *Mmm* localization inside the cytoplasm of endothelial cells. **G** The merging of fluorochromes highlighted the close interaction between *Mmm* (red color) and the alveolar macrophage (lysozyme, green color). Colocalization of red and green signals was shown in white (**H**) and indicated the localization of *Mmm* within cytoplasm of the alveolar macrophage. Nuclei were always stained in blue with DAPI. Scale bar: 2.5 µm (**G**, **H**), 5 µm (**A**–**D**), 7.5 µm (**E**, **F**).

DLIIF and LSCM investigations confirmed the *Mmm* localization within the above-mentioned tissues. In particular, LSCM demonstrated the presence of small amounts of *Mmm* within the cytoplasm of alveolar macrophages, of epithelial cells lining the bronchiolar and alveolar lumina, as well as of blood and lymphatic endothelial cells, both in challenged BREs (Figure 8) and in CBPP-naturally affected cattle (Figure 9).

### Survival and growth of *Mycoplasma mycoides* subsp. *mycoides* in bovine respiratory explants

**Figure 10:**
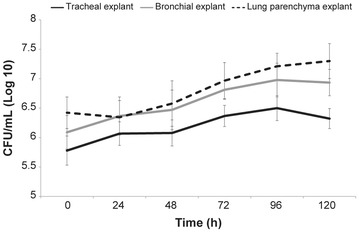
**Survival and growth of** ***Mmm*** **in challenged BREs.** Culture assays yielded the isolation of *Mmm* from all BREs, during the entire time course of the experiments. The *Mmm* titer slightly but significantly increased over time, reaching the highest levels in the lung explants. Data were represented as the mean ± SD.

Culture investigations demonstrated the survival of *Mmm* in all BREs, along the entire time course of the experiments. As shown in Figure 10, the titre of *Mmm* varied over time and reached the highest levels in the lung explants.

In tracheal explants, the *Mmm* titer significantly increased between T24 and T120, when compared with T0 (*p* ≤ 0.006); no significant difference was observed between T24 and T48 (*p* = 0.9), while a slight but significant reduction occurred between T96 and T120 (*p* = 0.04).

In bronchial explants, the *Mmm* titer significantly increased between T24 and T120, when compared with T0 (*p* ≤ 0.006); no significant difference was observed between T24 and T48 (*p* = 0.3), as well as between T72 and T120 (*p* ≥ 0.2).

In lung explants, the *Mmm* titer did not significantly change between T0 and T24 (*p* = 0.3); then, *Mmm* titer significantly increased between T48 and T96 (*p* ≤ 0.04), while no significant difference was observed between T96 and T120 (*p* = 0.3). At each time point, *Mmm* titer significantly varied between BREs (*p* < 0.05), except for lung *vs* bronchial explants at T24 (*p* = 0.9), T48 (0.5) and T72 (*p* = 0.2).

TCM collected from explant-containing wells proved to be negative after 72 hpi. Likewise, the *Mmm* titre in TCM alone (i.e. without explant) dramatically decreased and was no longer detectable after 72 h (Additional file 7).

### Gentamicin invasion assay on lung explants

**Figure 11:**
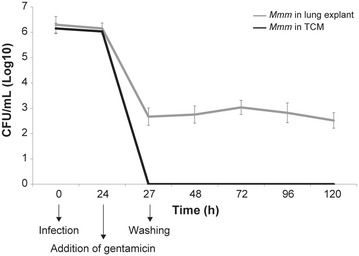
**Gentamicin invasion assays.** In lung explants, the *Mmm* titer significantly decreased at T27, after the treatment with gentamicin (*p* ≤ 0.0001). Then, the titer remained quite stable, with slight modifications at T72 (*p* = 0.02) and at T120 (*p* = 0.01). In TCM, culture tests proved to be negative from T27, after the treatment with gentamicin.

The gentamicin treatment sharply reduced the titre of *Mmm*, although it did not completely abolish its presence in lung explants. In TCM, *Mmm* was no longer detectable after the addition of gentamicin (Figure 11).

## Discussion

The pathogenesis of CBPP still shows a number of “dark sides”, mostly due to the lack of suitable study models. In fact, only few solid data are currently available about the early stages of infection, the selective involvement of specific cell types, as well as the molecular basis of cell and tissue damage [5, 6, 8, 17]. Considering the optimal preservation of cells and tissues, BREs may provide a relevant contribution to fill such gaps of knowledge and to minimize the use of experimental animals.

Explants have been successfully assessed to study the pathogenesis of viral respiratory diseases [23, 29–31] and, less frequently, of respiratory mycoplasmosis in both humans and animals [32–35]. Taken together, data reported herein confirm that explants can be easily obtained from slaughtered cattle [29] and indicate that BREs—particularly lung explants—provide the adequate microenvironment for *Mmm* survival and growth, closely resembling the in vivo habitat of this pathogen. As previously stated, severe changes of MALT were observed in BREs early on during the experiments, most likely due to the culture condition (i.e. the composition of the culture media). In fact, TCM for BREs lack fetal calf serum and other components, which are usually added to culture lymphoid tissues [36]. Moreover, TCM for lung explants contain glucocorticoids, which are known to induce the apoptosis of lymphocytes and probably worsened the changes of the MALT [37].

Adhesion is crucial for pathogenic mycoplasma to initiate infection [38]. However, no adhesins or host cell receptors have been identified so far for *Mmm* [8, 9]. Our results indicate that adhesion occurs in BREs. Differently from most respiratory mycoplasmas [34, 39–42], *Mmm* did not adhere to the ciliated epithelial cells lining the trachea and bronchi, whereas it quickly and considerably showed a specific tropism for the lower airways. Since CBPP spreads by the respiratory route, non-ciliated epithelial cells residing within bronchioles and alveoli should be regarded as the earliest and most relevant cellular targets of infection. As widely shown for other respiratory pathogens (namely, influenza viruses), the different expression of cellular surface molecules—which might vary with age and/or along the airways—could strongly influence the tropism of *Mmm* and should be investigated in depth [19, 43].

*Mmm* antigens were consistently detected inside the cytoplasm of phagocytic cells, as well as on and inside the endothelial cells of blood and lymphatic vessels. Overall, the IHC distribution pattern of *Mmm* in BREs largely overlapped that observed in CBPP naturally affected cattle [44–47], further supporting the suitability of BREs as an attractive and cost-effective model to study the early host–pathogen interaction.

Mycoplasmas have been generally considered extracellular parasites. However, the development of in vitro culture systems and the progress in imaging technologies led to reconsider such “dogma”. In fact, an increasing number of mycoplasmas have been shown to enter non-phagocytic host cells [28, 48–51]. The intracellular phase provides a protective niche for pathogens, in order to escape the immune defense and antimicrobial drugs [52]. Worthy of interest, we herein first demonstrated that *Mmm* is also able to penetrate inside non-phagocytic host cells. This *Mmm* feature could be of great relevance to the onset of chronic infections, which are crucial for the persistence of CBPP in cattle populations. The intracellular localization of *Mmm* was shown by two different, complementary approaches: LSCM and gentamicin invasion assay. The latter has been repeatedly used to verify the invasion and persistence of mycoplasmas inside eukaryotic cells [28, 53, 54] and was herein for the first time applied to explants, further strengthening our observation.

Some data suggest that pathogenicity of *Mmm* may result from toxic compounds (i.e. ROS), generated via glycerol metabolism and directly translocated inside the host cells [13, 18]. However, challenged BREs showed no evident pathological change, despite the abundant presence of *Mmm* in close contact with its target cells. These findings support the hypothesis that cell damage is not directly caused by mycoplasmas through the production of toxic metabolites, but is mediated by the immune and/or inflammatory response [48]. In this respect, we highlight that immune-mediated mechanisms (i.e. autoimmune reactions and type III hypersensitivity) might play a key role in the pathogenesis of vasculitis, which is a prominent and pathognomonic feature of natural and experimental CBPP [6, 14, 55].

In conclusion, the present study indicates that BREs well replicate the host tissue microarchitecture and provide a suitable substrate for *Mmm* survival and growth. We consider that respiratory explants from slaughtered cattle could act as useful tools to investigate the early host-*Mmm* interactions also fulfilling the principles of the three Rs stated by Russell and Burch [56]. In the near future, we consider that BREs could be successfully used to clarify some crucial and yet unknown steps of *Mmm* infection (e.g. to characterize the adhesins and their corresponding receptors on different cell types, to understand the mechanisms of *Mmm* entry inside the host cell, to investigate the early events of the inflammatory response, to provide new insights about the evolution of the antimicrobial resistance etc.). In this respect, we consider that similar models of study, developed in different animal species to investigate other respiratory pathogens [43], should be carefully regarded to open additional fields of investigation.

## Additional files

**Additional file 1.** **Composition of transport and culture media used for bovine respiratory explants.**

**Additional file 2.** **Technical details of the immunohistochemical protocols performed for selected cellular markers.**

**Additional file 3.** **Double-labelling indirect immunofluorescence (DLIIF) protocols.**

**Additional file 4.** **Representative photomicrographs of BREs.** The morphologic appearance of the tracheal (A–F) and bronchial (G–N) epithelium was well maintained for up to 120 hpc. Notably, the density of the cilia was preserved along the entire time course of the experiment. Likewise, the appearance of the underlying *lamina propria*—including the blood vessels—did not show any obvious change. The thickness and the dyeability of the alveolar walls slightly changed after the embedding with agarose gel (O vs P) and then remained almost unaltered up to 120 hpc. H&E staining. Scale bar: 50 µm (A–N), 100 µm (O–T).

**Additional file 5.** **Changes affecting the MALT in tracheal BREs.** At T0, no change affected the lymphoid tissue residing within the tracheal mucosa; the lymphoid cells were densely packed and showed a normal microscopic appearance (A). At T24, the tracheal MALT was depleted, with marked pyknosis and fragmentation of lymphoid cells (B). Such changes were more severe at T120 (C). Already at T24, the TUNEL assay (Tunel Apoptosis detection kit, Merck Millipore) demonstrated the presence of a very high number of apoptotic cells within the tracheal MALT (D). Moreover, few apoptotic cells were also seen within the tracheal epithelium and the upper layer of the *lamina propria*. Scale bar: 20 µm (A-C), 50 µm (D).

**Additional file 6.** **Immunohistochemistry for cellular markers in BREs.** A strong and specific IR for cytokeratins was evident within the cells of the tracheal epithelium and glands from a control tissue immediately fixed at the abattoir (A), as well as in an explant 120 hpc (B). Similarly, a strong and specific IR for vWF was seen within the endothelial cells of the trachea in a control tissue immediately fixed at the abattoir (C) and in an explant 120 hpc (D). Lysozyme-IR macrophages were detected within the tracheal *lamina propria* in a control tissue immediately fixed at the abattoir (E) and in an explant 120 hpc (F). Mayer’s hematoxylin counterstain. Scale bar: 100 µm (A–B), 50 µm (C–F).

**Additional file 7.** **Survival and growth of** ***Mmm*** **in TCM.** The graphic clearly shows that *Mmm* was not able to grow in TCM, its presence being no longer detected 72 h post-seeding. No significant difference was observed among different TCM (*p* ≥ 0.05).

## Abbreviations

CBPP: contagious bovine pleuropneumonia
*Mmm*: *Mycoplasma mycoides* subsp*. mycoides*
ROS: reactive oxygen species
BREs: bovine respiratory explants
BVDV: bovine viral diarrhea virus
BHV-1: bovine herpesvirus type 1
PI-3: parainfluenza-3 virus
BRSV: bovine respiratory syncytial virus
PCR: polymerase chain reaction
TM: transport medium
TCM: tissue culture medium
NBF: neutral buffered formalin
H&E: hematoxylin and eosin stain
IHC: immunohistochemistry
CFU: colony forming units
PBS: phosphate-buffered saline
DLIIF: double-labelling indirect immunofluorescence
LSCM: laser scanning confocal microscopy
vWF: von Willebrand factor
SD: standard deviation
MALT: mucosa-associated lymphoid tissue
IR: immunoreactivity
