# Supplementary material for: Respiratory explants as a model to investigate early events of contagious bovine pleuropneumonia infection
Source: Vet Res. 2018 Jan 12;49:5. doi: 10.1186/s13567-017-0500-z (PMC5766988; doi:10.1186/s13567-017-0500-z)
Supplement: Supplementary file 1 — Additional file 1. Composition of transport and culture media used for bovine respiratory explants. [file 13567_2017_500_MOESM1_ESM.docx]

**Additional file 1 Composition of transport and culture media used for bovine respiratory explants.**

| **Medium** | **Components** |
| --- | --- |
| Transport medium (TM) | 1. Sterile PBS, pH 7.2 2. Ampicillin (Sigma-Aldrich), 1 g/L 3. Amphotericin-B (Sigma-Aldrich), 2 mg/L |
| Tissue culture medium (TCM) for tracheal explants | 1. 500 mL DMEM (Gibco) 2. 500 mL RPMI (Gibco) 3. Glutamax (Gibco), 300 mg/L 4. Ampicillin, 300 mg/L (Sigma-Aldrich) 5. Amphotericin-B, 2 mg/L (Sigma-Aldrich) |
| Tissue culture medium (TCM) for bronchial explants | 1. 1,000 mL DMEM (Gibco) 2. Glutamax (Gibco), 300 mg/L 3. HEPES buffer 1M (Gibco), 20 mL/L 4. Ampicillin, 300 mg/L (Sigma-Aldrich) 5. Amphotericin-B, 2 mg/L (Sigma-Aldrich) |
| Tissue culture medium (TCM) for lung parenchyma explants | 1. 1000 mL DMEM (Gibco) 2. Glutamax (Gibco), 300 mg/L 3. Bovine insulin (Sigma-Aldrich), 1.5 mg/L 4. Hydrocortisone (Sigma-Aldrich), 0.3 mg/L 5. Vitamin A (Sigma-Aldrich), 0.5 mg/L 6. Ampicillin, 300 mg/L (Sigma-Aldrich) 7. Amphotericin-B, 2 mg/L (Sigma-Aldrich) |
