# Supplementary material for: Respiratory explants as a model to investigate early events of contagious bovine pleuropneumonia infection
Source: Vet Res. 2018 Jan 12;49:5. doi: 10.1186/s13567-017-0500-z (PMC5766988; doi:10.1186/s13567-017-0500-z)
Supplement: Supplementary file 2 — Additional file 2. Technical details of the immunohistochemical protocols performed for selected cellular markers. [file 13567_2017_500_MOESM2_ESM.docx]

**Additional file 2 Technical details of the immunohistochemical protocols performed for selected cellular markers.**

| **Primary antibody** | **Clone** | **Cellular targets** | **Manufacturer** | **Final dilution** | **Antigen retrieval** | **Visualization system** | **Control tissues** |
| --- | --- | --- | --- | --- | --- | --- | --- |
| von Willebrandt Factor | // | Endothelial cells, platelets | Dako | 1:800 | Enzymatic digestion with trypsin (0.01% in 0.15 M Tris-HCl buffer, pH 7.8) | Dako REAL^TM^ detection system | Tracheal, bronchial and pulmonary samples, promptly fixed in 10% NBF at the abattoir |
| Lysozyme | // | Monocyte-macrophage system | Dako | 1:1000 |  |  |  |
| Cytokeratins | AE1/AE3 | Epithelial cells | Dako | 1:250 |  |  |  |
