# Supplementary material for: Respiratory explants as a model to investigate early events of contagious bovine pleuropneumonia infection
Source: Vet Res. 2018 Jan 12;49:5. doi: 10.1186/s13567-017-0500-z (PMC5766988; doi:10.1186/s13567-017-0500-z)
Supplement: Supplementary file 3 — Additional file 3. Double-labelling indirect immunofluorescence (DLIIF) protocols. [file 13567_2017_500_MOESM3_ESM.docx]

**Additional file 3 Double-labelling indirect immunofluorescence (DLIIF) protocols***

| **Primary antibodies** | **Final dilution** | **Incubation** |
| --- | --- | --- |
| Rabbit polyclonal anti-*Mmm*** | 1:1280 | Overnight at 4 °C |
| Murine monoclonal anti-*Mmm*** | 1:10 | Overnight at 4 °C |
| Anti-cytokeratins*** | 1:250 | Overnight at 4 °C |
| Anti-von Willebrand factor*** | 1:400 | Overnight at 4 °C |
| Anti-lysozyme*** | 1:500 | Overnight at 4 °C |
| **Biotinylated secondary**  **Antibodies** | **Final dilution** | **Incubation** |
| Goat anti-mouse IgG  (Vector Laboratories, Inc.) | 1:200 | 30 min at room  temperature |
| Goat anti-rabbit IgG  (Vector Laboratories, Inc.) | 1:200 | 30 min at room  temperature |
| **Fluorochromes** | **Final dilution** | **Incubation** |
| Texas red avidin DCS  (Vector Laboratories, Inc.) | 1:200 | 15 min at room  temperature |
| Fluorescein avidin DCS  (Vector Laboratories, Inc.) | 1:200 | 15 min at room  temperature |

*Rabbit and murine primary antibodies were combined in each DLIIF run;

**Anti-*Mmm* primary antibodies were manufactured at the *Istituto Zooprofilattico Sperimentale dell’Abruzzo e Molise “G. Caporale”*, Teramo, Italy;

***see Additional file 2 for further technical details.
