# Supplementary figures and images for: Respiratory explants as a model to investigate early events of contagious bovine pleuropneumonia infection
Source: Vet Res. 2018 Jan 12;49:5. doi: 10.1186/s13567-017-0500-z (PMC5766988; doi:10.1186/s13567-017-0500-z)

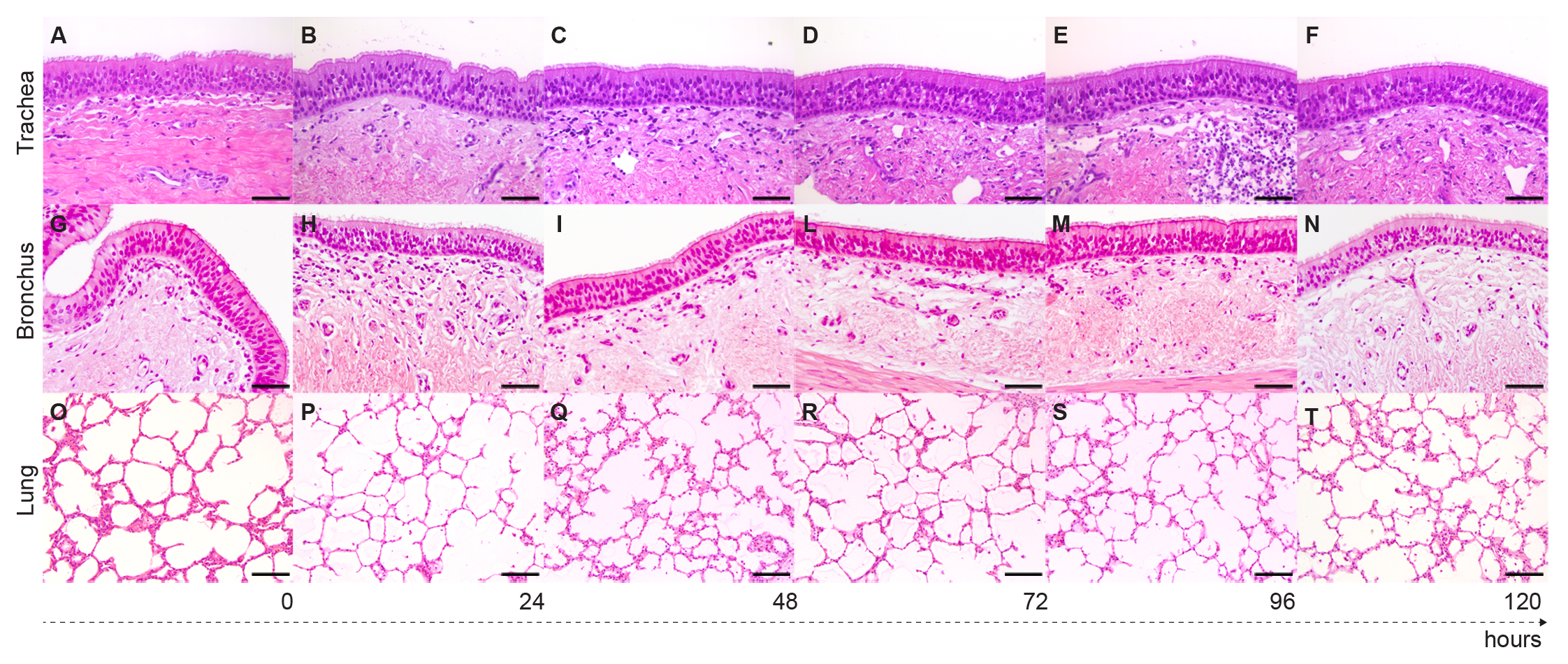

Supplement: Supplementary file 4 — Additional file 4. Representative photomicrographs of BREs. The morphologic appearance of the tracheal (A–F) and bronchial (G–N) epithelium was well maintained for up to 120 hpc. Notably, the density of the cilia was preserved along the entire time course of the experiment. Likewise, the appearance of the underlying lamina propria—including the blood vessels—did not show any obvious change. The thickness and the dyeability of the alveolar walls slightly changed after the embedding with agarose gel (O vs P) and then remained almost unaltered up to 120 hpc. H&E staining. Scale bar: 50 µm (A–N), 100 µm (O–T). [file 13567_2017_500_MOESM4_ESM.tif]

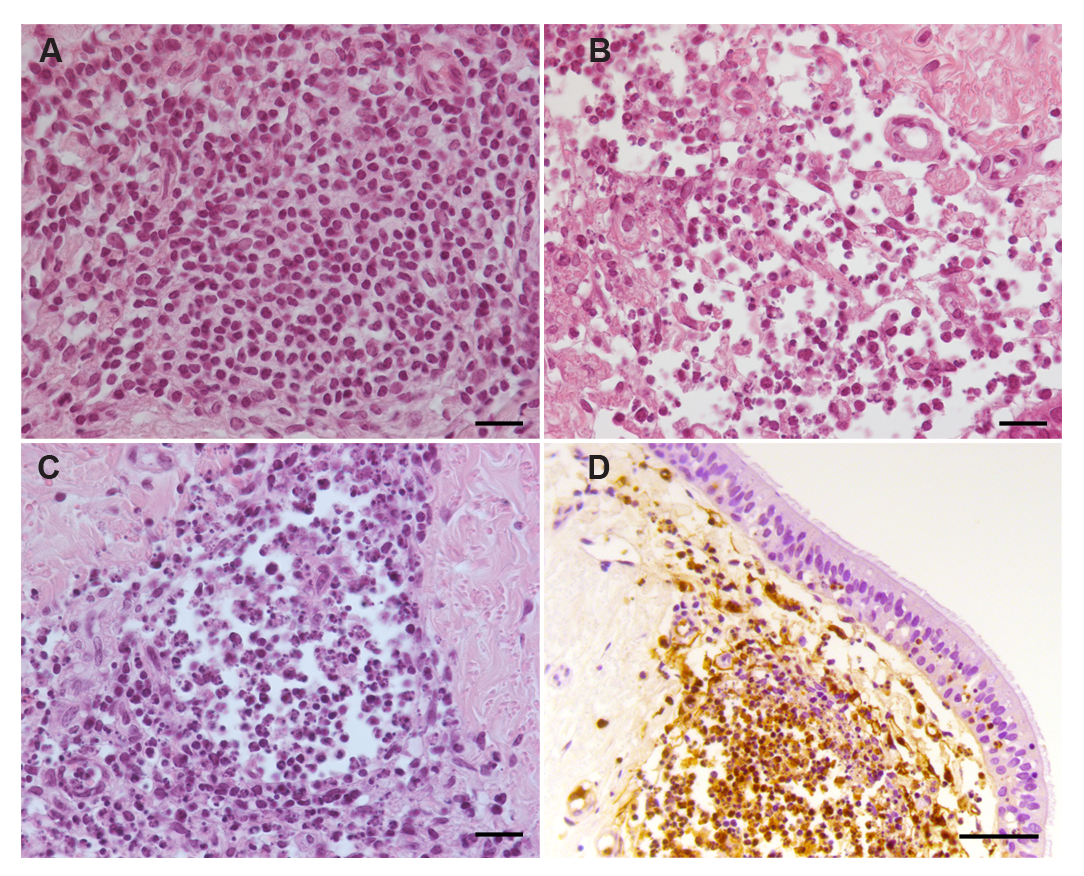

Supplement: Supplementary file 5 — Additional file 5. Changes affecting the MALT in tracheal BREs. At T0, no change affected the lymphoid tissue residing within the tracheal mucosa; the lymphoid cells were densely packed and showed a normal microscopic appearance (A). At T24, the tracheal MALT was depleted, with marked pyknosis and fragmentation of lymphoid cells (B). Such changes were more severe at T120 (C). Already at T24, the TUNEL assay (Tunel Apoptosis detection kit, Merck Millipore) demonstrated the presence of a very high number of apoptotic cells within the tracheal MALT (D). Moreover, few apoptotic cells were also seen within the tracheal epithelium and the upper layer of the lamina propria. Scale bar: 20 µm (A-C), 50 µm (D). [file 13567_2017_500_MOESM5_ESM.tif]

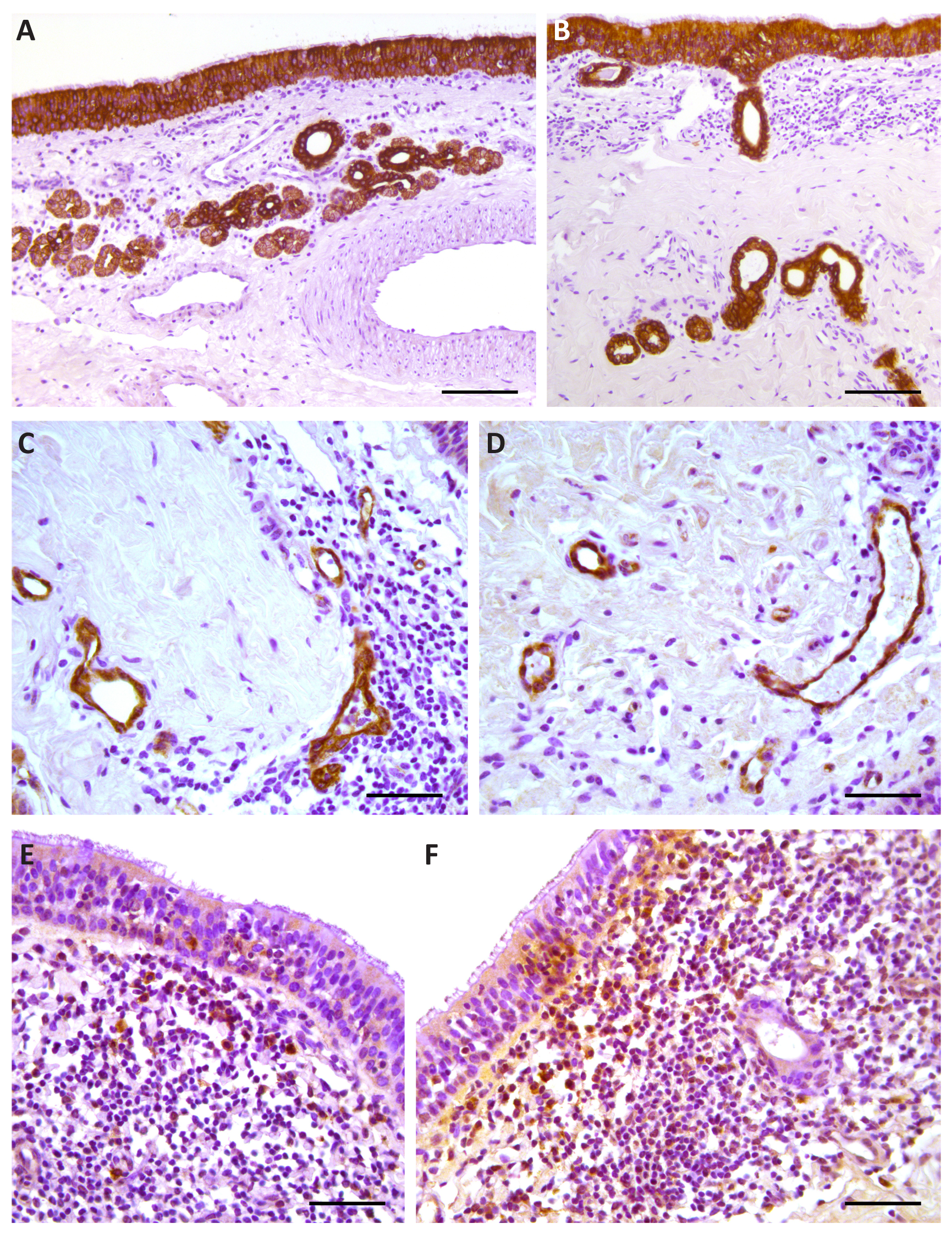

Supplement: Supplementary file 6 — Additional file 6. Immunohistochemistry for cellular markers in BREs. A strong and specific IR for cytokeratins was evident within the cells of the tracheal epithelium and glands from a control tissue immediately fixed at the abattoir (A), as well as in an explant 120 hpc (B). Similarly, a strong and specific IR for vWF was seen within the endothelial cells of the trachea in a control tissue immediately fixed at the abattoir (C) and in an explant 120 hpc (D). Lysozyme-IR macrophages were detected within the tracheal lamina propria in a control tissue immediately fixed at the abattoir (E) and in an explant 120 hpc (F). Mayer’s hematoxylin counterstain. Scale bar: 100 µm (A–B), 50 µm (C–F). [file 13567_2017_500_MOESM6_ESM.tif]

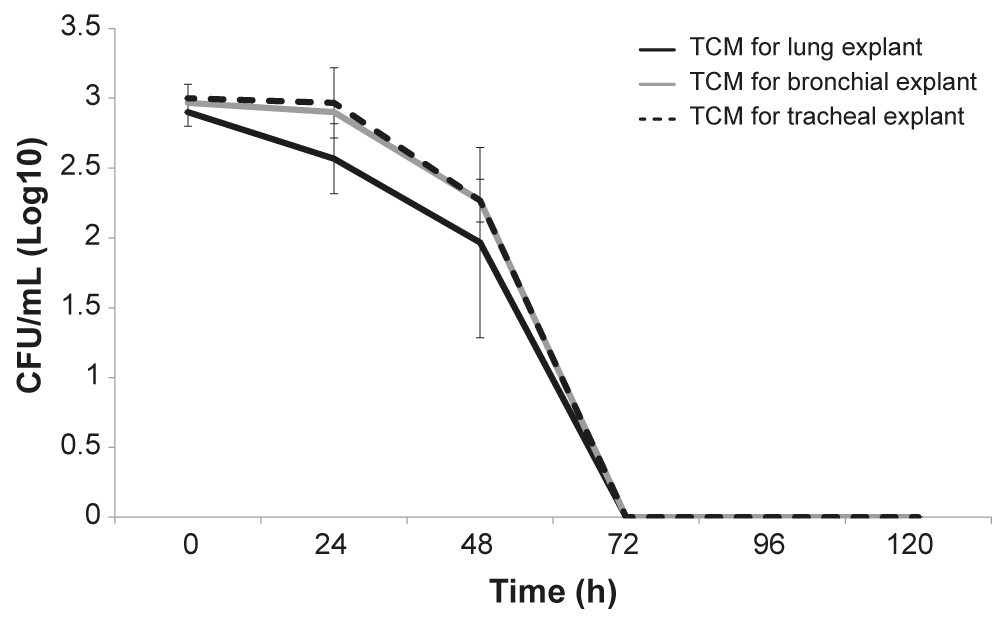

Supplement: Supplementary file 7 — Additional file 7. Survival and growth of Mmm in TCM. The graphic clearly shows that Mmm was not able to grow in TCM, its presence being no longer detected 72 h post-seeding. No significant difference was observed among different TCM (p ≥ 0.05). [file 13567_2017_500_MOESM7_ESM.tif]
